# Inhibitory Role of the Mud Crab Short Neuropeptide F in Vitellogenesis and Oocyte Maturation via Autocrine/Paracrine Signaling

**DOI:** 10.3389/fendo.2018.00390

**Published:** 2018-07-13

**Authors:** Chenchang Bao, Yanan Yang, Huiyang Huang, Haihui Ye

**Affiliations:** ^1^College of Ocean and Earth Sciences, Xiamen University, Xiamen, China; ^2^Fujian Collaborative Innovation Center for Exploitation and Utilization of Marine Biological Resources, Xiamen, China

**Keywords:** short neuropeptide F, ovary, oocyte maturation, vitellogenesis, crustacean

## Abstract

Neuropeptides, in addition to their classical role in the nervous system, act on intraovarian factors to regulate reproductive functions in vertebrates. However, this function of neuropeptides has not been characterized in crustaceans. Short neuropeptide F (sNPF), a highly conserved invertebrate neuropeptide, has been reported to be involved in feeding, metabolism, and in differentiation processes including reproduction. Although sNPF and its receptor (sNPFR) have been detected in the ovary in different species, ovarian colocalization of sNPF/sNPFR has not been investigated. In this study, we identified *Scylla paramamosain* (mud crab) sNPF (*Sp*-sNPF) as an endogenous ligand for the *S. paramamosain* orphan G protein-coupled receptor NPY2R in mammalian cell line HEK293T. We designated this receptor as *Sp-sNPFR*. RNA *in situ* hybridization in pre-vitellogenic ovary and reverse transcription-PCR on isolated denuded oocytes and follicle layers showed that *Sp-sNPF* was exclusively localized to the follicle cells, whereas *Sp-sNPFR* was detected in both follicle cells and oocytes. We also found that *Sp*-sNPF partly suppressed spontaneous maturation of denuded oocytes and caused intracellular cAMP accumulation and Ca^2+^ mobilization. Moreover, injection of synthetic *Sp*-sNPF peptides inhibited the expression of vitellogenin and vitellogenin receptor genes *in vivo*. These combined results suggest for the first time that *Sp*-sNPF may have inhibitory functions in vitellogenesis and oocyte maturation possibly via the autocrine/paracrine pathway.

## Introduction

Neuropeptides are widespread in the nervous system of animals (1). Neuropeptides are secreted from neural cells and act as neuromodulators or neurotransmitters in the nervous system (2). Furthermore, neuropeptides are secreted from neuroendocrine cells, transported through blood to peripheral organs, and function as neurohormones (3). They also play a pivotal role in regulating reproduction (4). One such example is that of gonadotropin-releasing hormone (GnRH), a key decapeptide hormone involved in the regulation of reproduction which is secreted by the hypothalamus and transported via the hypophysial portal system to stimulate the production of luteinizing hormone (LH) and follicle-stimulating hormone (FSH), which further act on the ovary (5, 6). In addition to its involvement in this classical hypothalamic-pituitary-gonadal (HPG) reproduction axis in vertebrates, GnRH also plays a role as a neuroendocrine factor by directly binding to its receptor (GnRHR) in the ovary (7, 8). Mostly, neuropeptides are released by the nervous system and regulate reproduction directly or indirectly (8, 9). However, neuropeptides can also act as intraovarian regulatory factors. Intraovarian GnRH/GnRHR system has been suggested to play an autocrine/paracrine role in gonadal steroidogenesis, cell proliferation, and apoptosis (8, 10–13). In addition to GnRH/GnRHR, two key upstream regulation neuropeptide systems, gonadotrophin-inhibitory hormone (GnIH)/GnIH receptor (GnIHR) and kisspeptin/kisspeptin receptor, in the vertebrate reproduction axis also exist in ovary (14–23). The regulation of intraovarian neuropeptides has been well studied in vertebrates (7, 8, 18, 24, 25). However, a detailed knowledge about the intraovarian neuropeptides acting on ovary in invertebrates has not been reported.

Short neuropeptide F (sNPF), a homolog of vertebrate neuropeptide Y (NPY), is a C-terminally conserved neuropeptide in invertebrates. sNPF belongs to FMRFa-like peptides (FLPs) with the RFamide conserved motif (26) and has been widely studied in insects, especially in *Drosophila* (27). The first sNPF isolated from the midgut of American cockroach, *Periplaneta americana*, was postulated to play a role in digestion (28). Studies have mainly focused on the role of sNPF in regulation of feeding and metabolism (29–34). However, a role for sNPF in reproduction has also been identified. For instance *Led*-NPF-1, sNPF peptide from *Leptinotarsa decemlineata* (Colorado potato beetle) and *Scg*-NPF, a *Locusta migratoria* sNPF peptide, were shown to accelerate egg development in *L. migratoria* (34–37). However, their regulatory role in signaling is not known. *Drosophila* sNPF and its receptor (sNPFR) have been considered as upstream regulators of insulin signaling pathway (30). In *Drosophila*, sNPF and sNPFR1 proteins were detected in the larval brain where sNPFR1 was localized to the brain insulin producing cells (IPCs) and sNPFnergic neurons were found adjacent to these IPCs (30, 38). sNPF peptides stimulate insulin-like peptides (ILPs) synthesis in IPCs and affect insulin signaling (30). In insects, insulin signaling pathway regulates developmental and differentiation functions such as reproduction (39, 40). Juvenile hormone (JH) and 20-hydroxyecdysone (20E) were found to act as the insect gonadotropic hormones and to be regulated by insulin signaling by a complex physiological regulatory network (39, 40). sNPF peptides can directly regulate the production of JH. In the silkworm, *Bombyx mori*, sNPF peptides localize to the corpora cardiaca and inhibit JH biosynthesis in the corpora allata (41, 42). Moreover, sNPF was speculated as a neurohormone directly functioning on the ovary of red imported fire ant, *Solenopsis invicta* Buren as the *S. invicta* sNPF receptor (*Si*-sNPFR) protein was detected in oocytes by immunolocalization (43). These findings suggest that sNPF may serve as neuro-regulator in reproduction. Whether or not sNPF directly stimulates ovary is not known.

Recently, with the rapid development of methodologies, such as next-generation sequencing and mass spectrometry, many sNPF peptides have been identified in various crustacean species including the mud crab (*Scylla paramamosain*) (44–50). However, the reproductive function of sNPF in crustaceans is not known. *S. paramamosain* sNPF (*Sp*-sNPF) transcript was identified to encode a 126 amino acids (aa) precursor peptide with a 25 aa signal peptide followed by three mature peptides, each 9–12 aa in length and each containing a XPXRLRFamide (X represent variable residues) conserved motif (44). Interestingly, in our previous study, we found that *Sp*-sNPF transcript was highly expressed in the ovary in addition to the nervous system (44). We hypothesized that sNPF may act as intraovarian regulatory factor similar to vertebrate neuropeptides. In this study, we investigated the potential intraovarian autocrine/paracrine role of *Sp*-sNPF by receptor identification, ligand-receptor localization, signal transduction, and by biological relevance.

## Materials and methods

### Animals

Crabs were purchased in the eighth market in Xiamen, Fujian Province, China. According to histological characteristics (51) and gonadosomatic index (GSI) (52), ovarian development was classified into five stages: undeveloped stage (stage I, GSI = 0.57 ± 0.47), pre-vitellogenic stage (stage II, GSI = 2.19 ± 0.21), early vitellogenic stage (stage III, GSI = 3.68 ± 0.20), late vitellogenic stage (stage IV, GSI = 7.81 ± 0.94), and mature stage (stage V, GSI = 10.49 ± 0.49). Anesthesia on ice was administered before dissections. The study was approved by Xiamen University animal care committee.

### Molecular cloning and plasmid construction

Total RNA from the crab ovary was isolated with the Trizol Reagent (Invitrogen) according to the manufacturer's instructions. The cDNA was synthetized using the RevertAid First Strand cDNA Synthesis Kit (Thermo) as per manufacturer's instructions. To clone the entire coding region of *Sp*-sNPFR gene, primers (Table 1) were designed based on the sequence in the transcriptome databases of brain (SRA accession: SRP068003) and muscle (SRA accession: SRP111448) from *S. paramamosain*. PCR product was sequenced prior to inserting into the HindIII and BamHI sites of the pEGFP-N1 vector. The construct was sequenced to validate the sequence and orientation.

**Table 1:** Primers used for plasmid construction, real-time PCR and *in situ* hybridization probe preparation.

**Primers and Probes**	**Sequences**
**PRIMERS FOR** ***SP*****-SNPFR-EXPRESSING PLASMID CONSTRUCTION**	
*Sp*-sNPFR-ORF-F	5′-CCCAAGCTTGCCACCATGATGGGCTCGAGCACCTTAC-3′
*Sp*-sNPFR-ORF-R	5′-CGCGGATCCCGCACATACTCGGACGCCTCGC-3′
**PRIMERS FOR** ***IN SITU*** **HYBRIDIZATION PROBE PREPARATION**	
*Sp*-sNPF probe-F	5′-ATGGGCGTGAACGGCGT-3′
*Sp*-sNPF probe-R	5′-TTACTGCTCCTGGCTGACCATGT-3′
*Sp*-sNPFR probe-F	5′-GAGGCGTCCGAGTATGTGTGAA-3′
*Sp*-sNPFR probe-R	5′-CATTTGATGTCGTAAAGCTGAAGTG-3′
**PRIMERS FOR REAL-TIME PCR**	
*Sp*-sNPF real-time-F^*^	5′-GGTGACTCCGATTTAATGCTTT-3′
*Sp*-sNPF real-time-R^*^	5′-TGGCTTCCACTGCCGCTA-3′
*Sp*-sNPFR real-time-F	5′-CAAGTGCTAACCGCTGTAAAATG-3′
*Sp*-sNPFR real-time-R	5′-CGTCCAGGATGGTGTTTAGTGA-3′
*Sp*-Vg quantitative real-time-F^**^	5′-GAGTGATGATGGAGGTGTCCTG-3′
*Sp*-Vg quantitative real-time-R^**^	5′-GACCTTGAGCGATTCTGGTGACGA-3′
*Sp*-VgR quantitative real-time-F^***^	5′-TTCTATACCAGGCCACTACC-3′
*Sp*-VgR quantitative real-time-R^***^	5′-TTTTCACTCCAAGCACACTC-3′
β-actin-F^**^	5′-GAGCGAGAAATCGTTCGTGAC-3′
β-actin-R^**^	5′-GGAAGGAAGGCTGGAAGAGAG-3′

*ORF, open reading frame; F, forward; R, reverse*.

* Represent these primers cited from Bao et al. (44)

** represent these primers cited from Gong et al. (53)

*** *represent these primers cited from Shu et al. (54)*.

### Cell culture and transient transfection

Human embryonic kidney cell lines (HEK293T) were maintained in high glucose DMEM (Hyclone) supplemented with 10% fetal bovine serum (Gibco), 100 U/mL penicillin and 100 μg/mL streptomycin (Hyclone) at 37°C in a humidified incubator (Thermo) containing 5% CO_2_. Cells were seeded overnight in 6-well plates and transiently co-transfected with 1 μg of the *Sp*-sNPFR/pEGFP-N1 plasmid, 0.8 μg of the reporter gene pCRE-luc, and 0.4 μg of internal control gene pRL-TK using 5 μL Lipofectamine 2000 (Invitrogen) reagent following the manufacturer's protocols. Empty pEGFP-N1 plasmid was used for mock transfection.

### Confocal microscopy

For detection of sNPFR expression on the cell surface, HEK293T transiently transfected with *Sp*-sNPFR/pEGFP-N1 plasmid for 24 h were stained with the cell membrane probe DiI (Beyotime) at 37°C for 10 min and fixed with 4% paraformaldehyde (PFA) for 15 min. Cells were further incubated with a nuclear dye DAPI (Invitrogen) for 10 min. Stained cells were mounted in 50% glycerol and imaged using a two-photon laser confocal fluorescence microscopy (LSM 780 NLO, Zeiss).

### Activation of signaling

HEK293T transiently co-transfected with *Sp*-sNPFR/pEGFP-N1 and dual-luciferase reporter system vectors were grown to 90% confluency in a 24-well plate. Cells were first treated with synthetic *Sp*-sNPF peptide mixture and the mixture containing 9 synthetic FLPs (GL Biochem, Table 2) at 1 μM each. Further, various concentrations of individual peptides were used to record dose-dependent curves. After incubation for 6 h, ligand-induced changes in luciferase activity were measured by GloMax® 20/20 Luminometer (Promega) with Dual-Luciferase® Reporter Assay System Kit (Promega).

**Table 2:** Sequences of synthetic peptides for binding tests.

**Peptides**	**Sequence**	**Peptides**	**Sequence**
**Mixture-1**		**Mixture-2**	
*Sp*-sNPF1	APPSMRLRF-NH2	*Sp*-FLRFamide-1	GYSKNYLRF-NH2
*Sp*-sNPF2	SMPTLRLRF-NH2	*Sp*-FLRFamide-4/9	DRNFLRF-NH2
*Sp*-sNPF3	KDARTPALRLRF-NH2	*Sp*-FLRFamide-5	SGHRNYLRF-NH2
		*Sp*-FLRFamide-8	GYNRSFLRF-NH2
		*Sp*-NPF1_56−66_	YFAIAGRPRF-NH2
		*Sp*-NPF2_59−69_	IYSHMTRPRF-NH2
		*Sp*-FMRFamide	FMRF-NH2
		*Sp*-sulfakinin-1	EFDDY_(SO3H)_GHMRF-NH2
		*Sp*-myosuppressin	pQDLDHVFLRF-NH2

*All peptides were purified by HPLC and the purity is over 95%. -NH2 indicates C-terminal amidation. pQ indicates N-terminal pyroglutamylation. Y_(SO3H)_ indicates Tyrosine sulfate*.

### *In situ* hybridization

Crab ovaries in a pre-vitellogenic stage were dissected, fixed in 4% PFA, and prepared for *in situ* hybridization as described (53). Primers, listed in Table 1, spanning 381 and 250 bp nucleotides of *Sp-sNPF* and *Sp-sNPFR* cDNA, respectively, were used for the preparation digoxigenin-labeled cRNA riboprobes using a DIG-RNA Labeling Kit (Roche). Hybridization was performed at 57°C for 16 h according to the manufacturer's instructions.

### Isolation and incubation of crab ovary

Ovaries (late vitellogenic stage) with fully grown (FG) oocytes were removed from female crabs and placed in 10 cm culture dish containing calcium-free crab saline (440 mM NaCl, 11.3 mM KCl, 26 mM MgCl_2_, 10 mM Hepes, 10 mM Glucose, pH 7.4 adjusted with NaOH) (55). The follicle layers were carefully separated with fine forceps under stereoscope (Leica). To determine the expression of *Sp-sNPF* and *Sp-sNPFR* gene, total RNA from intact ovary, denuded oocytes, and follicle layers were extracted with the Trizol Reagent (Invitrogen). cDNA was synthesized using TransScript II One-Step gDNA Removal and cDNA Synthesis SuperMix kit (TransGen Biotech) according to the manufacturer's protocols. The cDNA templates were used for PCR amplification using *Sp-sNPF* and *Sp-sNPFR* primers (Table 1). PCR products were analyzed by 1.5% agarose gel electrophoresis.

### Intracellular calcium measurement

The denuded oocytes were washed thrice after they were separated from the ovaries with FG oocytes and were loaded with 1 μM Fluo-4 AM (Molecular Probes) for 40 min in 25°C. Oocytes were washed thrice, resuspended in calcium-free crab saline, and incubated for 30 min at 25°C. Calcium flux was measured in oocytes stimulated with *Sp*-sNPF peptides using excitation at 486 and emission at 526 nm in a fluorescence spectrometer (Infinite 200Pro, Tecan).

### Effect of *Sp*-sNPFs on oocyte maturation

To obtain spontaneously matured oocytes, ovaries with the oocytes in germinal vesicle (GV) stage were placed in a glass dish with crab serum (the status of GV stage oocytes was maintained better in crab serum than that in crab saline, data not shown). After the follicle layers were removed, the denuded oocytes were isolated from ovary and incubated in crab serum containing 5 μM *Sp*-sNPF peptides. The observation of oocyte germinal vesicle breakdown (GVBD) was carried out as described (56). The GVBD was counted in intact ovary as well as in the denuded oocytes following incubation with in *Sp*-sNPF peptides for 0, 5, 15, 30, and 60 min.

### Measurement of the accumulation of cAMP

The measurement of cAMP accumulation and the calculation of GVBD rate were performed at the same time. Oocytes were lysed and the concentration of cAMP was determined using a competitive binding technique based on enzyme-linked immunosorbent assay (ELISA) (cAMP EIA kit, Cayman) according to the manufacturer's protocols.

### *In vivo* effect of *Sp-*sNPF peptides on *Sp-Vg* and *Sp-VgR* expression

Female crabs in pre-vitellogenic stage (weighing 45–56 g) were reared in the laboratory and were fed a shrimp diet. The initial control crabs were sacrificed on first day of the injection assay. Ten microliter of 1 mM *Sp*-sNPF peptides (final concentration in hemolymph about 5 μM) were injected into the arthrodial membrane at the base of last walking leg every 2 days while concurrent control crabs received the same volume of carrier. After 10 days, crabs were dissected and RNA extracted. cDNA was synthesized from hepatopancreas and ovaries and used in quantitative real-time PCR (qRT-PCR) to detect the expression level of *vitellogenin* (*Sp-Vg*) and *vitellogenin receptor* (*Sp-VgR*). The qRT-PCR was performed in the 7500 Fast Real-Time PCR system (Applied Biosystems) with SYBR Premix Ex Taq (TaKaRa) as following protocol: 95°C for 30 s, followed by 40 cycles at 95°C for 5 s, 60°C for 30 s, and 72°C for 30 s.

### Data analysis

All values are expressed as mean ± SD. Multiple sequence alignment of sNPFR sequences was performed with ClustalX and embellished by LaTEX TexShade (57). Data of ligand-receptor assays were analyzed using nonlinear curve fitting (GraphPad Prism 6.0) to obtain activation curves and EC_50_ values. The mRNA levels of *Sp-Vg* and *Sp-VgR* gene were normalized against the internal control β*-actin*, and the relative expression was calculated using the 2^−ΔΔ*Ct*^ method. Statistical significance was determined using one-way ANOVA followed by Duncan's multiple range tests. All statistical analyses were performed using IBM SPSS Statistics 20.0. Pictures were drawn by GraphPad Prism 6.0 and modified with Adobe Photoshop CS5.

## Results

### Cloning and expression of *Sp*-sNPFR in HEK239T cells

**Figure 1:**
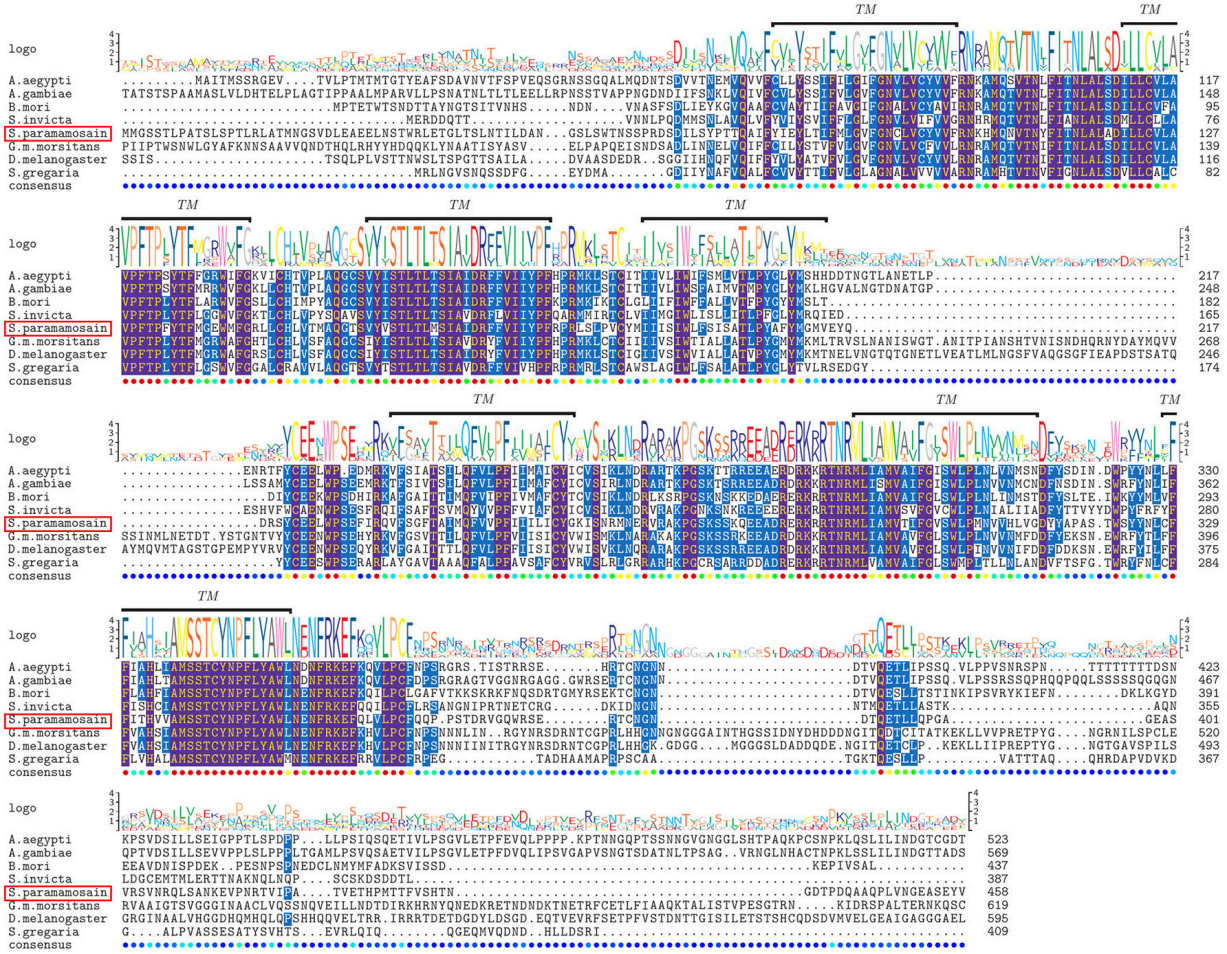
Comparative sequence alignment of sNPF receptors in different animals. Protein sequence alignment of sNPF receptors for *S. paramamosain* with *Aedes aegypti, Anopheles gambiae, Bombyx mori, Solenopsis invicta, Glossina morsitans morsitans, Drosophila melanogaster* and *Schistocerca gregaria*. The transmembrane helices are indicated by TM. Sequence logo is shown above alignments.

**Figure 2:**
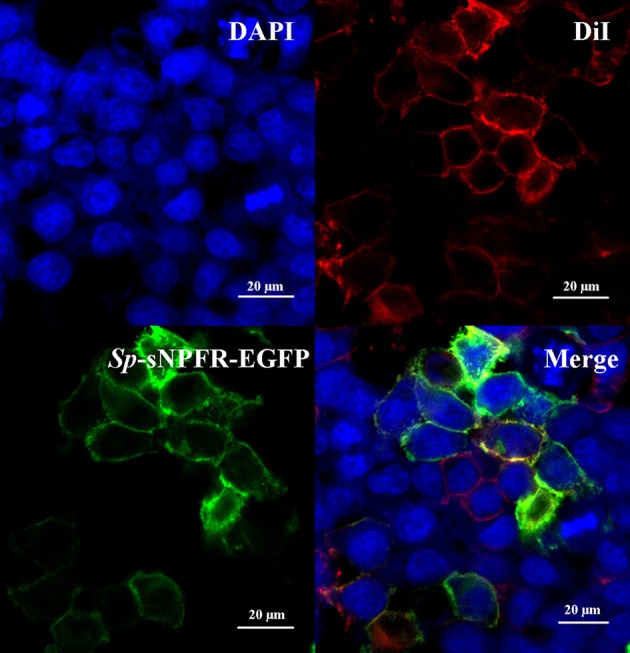
Expression of *Sp*-sNPFR in HEK293T. HEK293T were transiently co-transfected with *Sp*-sNPFR/pEGFP-N1, pCRE-luc and pRL-TK. Cells expressing *Sp*-sNPFR-EGFP fusion protein were stained with a nuclei probe (DAPI) and a membrane plasma probe (DiI).

Two partial sequences annotated as neuropeptide Y receptor type 2 (NPY2R) were mined from two transcriptome databases of *S. paramamosain*. The predicted *Sp-sNPFR* gene sequence (GenBank accession number: MH382826) encoding 458 amino acids was obtained when the two partial sequences were assembled. The protein sequence showed 60.1% identity to the mosquito, *Aedes aegypti* sNPF receptor, 61.1% identity to the African malaria mosquito, *Anopheles gambiae* sNPF receptor, 58.7% identity to *B. mori* sNPF receptor, 56.4% identity to *S. invicta* sNPF receptor, 61.9% identity to the tsetse fly, *Glossina morsitans morsitans* sNPF receptor, 52.4% identity to the fruit fly, *Drosophila melanogaster* sNPF receptor, and 54.4% identity to the Desert Locust, *Schistocerca gregaria* sNPF receptor (Figure 1). Enhanced green fluorescent protein (EGFP) fused to the C-terminus of *Sp*-sNPFR was transiently expressed in HEK293T. Confocal microscopy showed that *Sp*-sNPFR-EGFP mainly localized to the cell membrane as demonstrated (Figure 2).

### Activation of *Sp*-sNPFR by *Sp*-sNPFs

**Figure 3:**
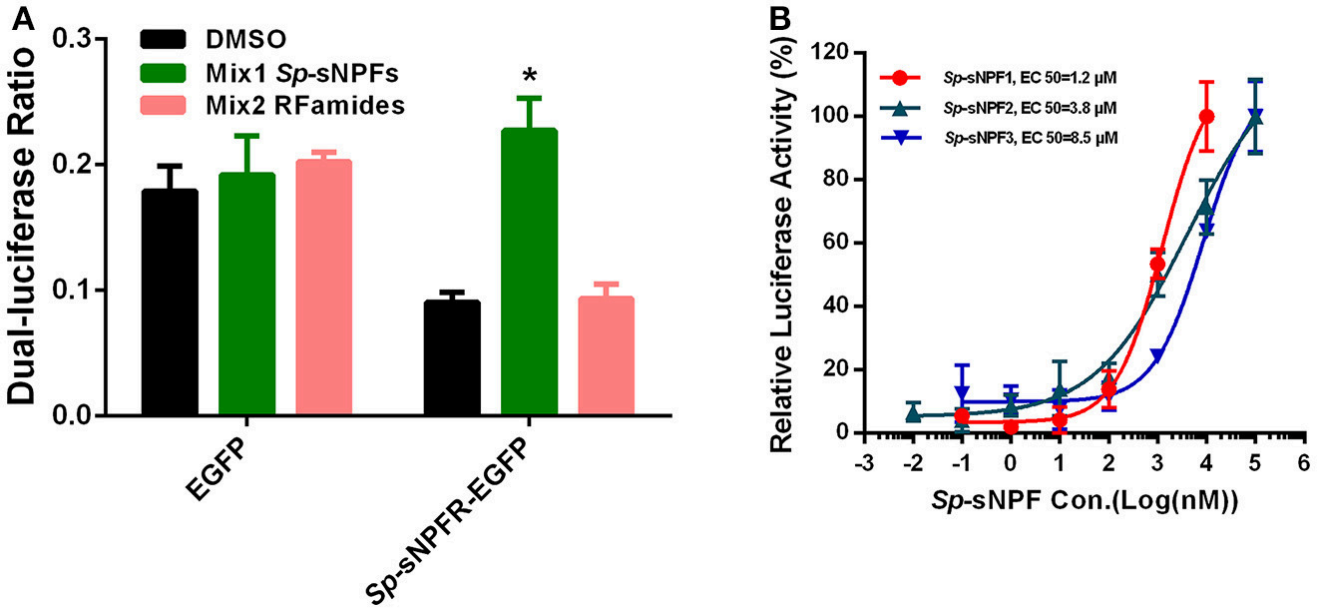
Ligand-receptor activation of signaling. **(A)** Relative luciferase activity of *Sp*-sNPFR-EGFP expressing cells after exposure to two peptide mixutures with a fixed concentration of 1 μM each peptide (*P < 0.05). **(B)** HEK293T transiently co-transfected with *Sp*-sNPFR/pEGFP-N1, pCRE-luc, and pRL-TK were treated with different doses of *Sp*-sNPF1, *Sp*-sNPF2, and *Sp*-sNPF3 and the concentration of cAMP was determined. Values are plotted as mean ± SD from three biological replicates.

We found that in cells transfected with *Sp*-sNPFR and dual-luciferase reporter, *Sp*-sNPFR was exclusively activated by *Sp*-sNPF peptides mixture, while all other FLPs with the RFamide C-terminus did not activate *Sp*-sNPFR at 1 μM concentration (Figure 3A). Furthermore, *Sp*-sNPFR was found to be activated by all the three *Sp*-sNPF peptides in a dose-dependent manner. The EC_50_ values were found to be 1.2, 3.8, and 8.5 μM for *Sp*-sNPF1, *Sp*-sNPF2, and *Sp*-sNPF3, respectively (Figure 3B).

### Localization of *Sp-sNPF* and *Sp-sNPFR* mRNA in the ovary

**Figure 4:**
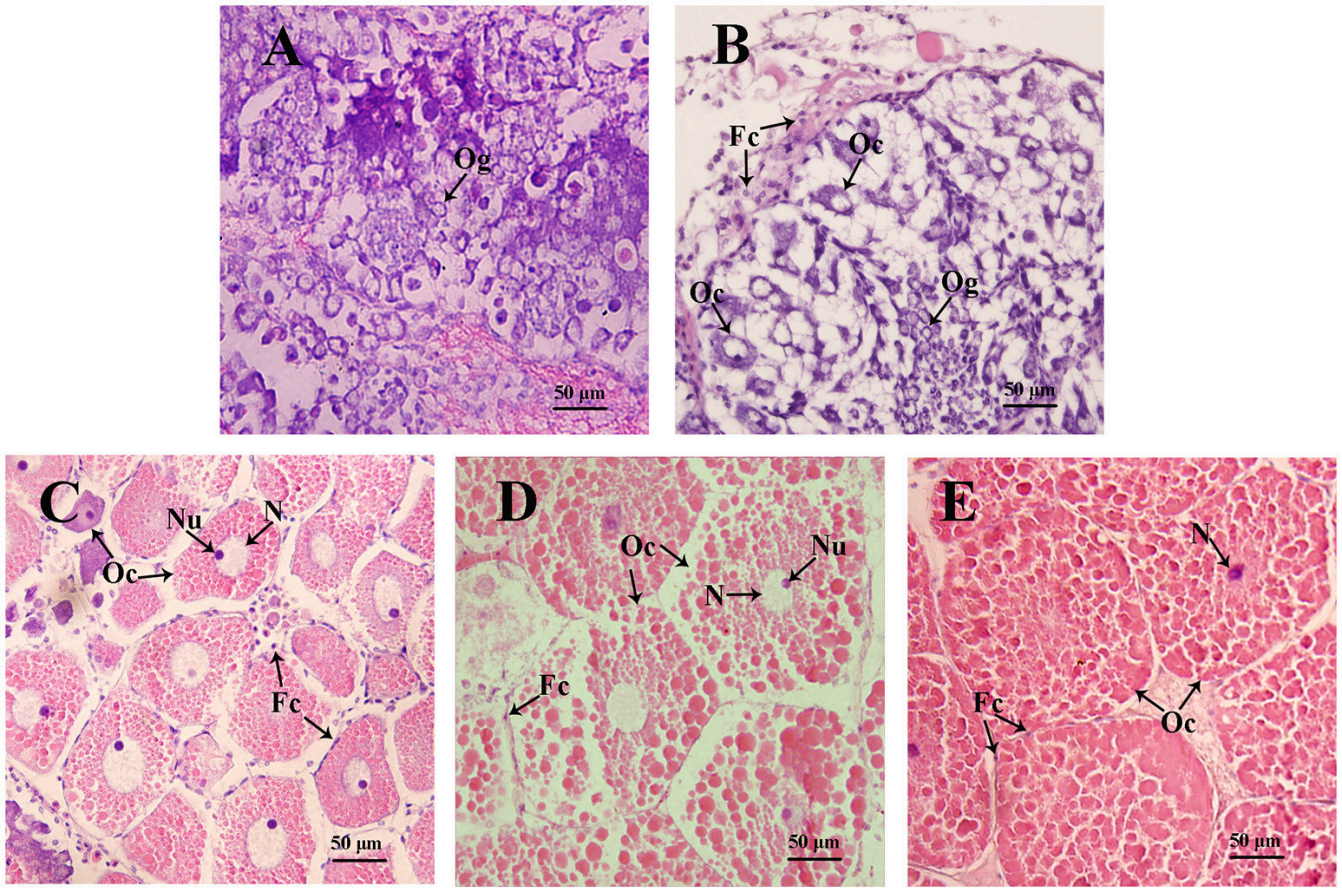
Sections of ovaries from various stages of ovarian development in *S. paramamosain*. **(A)** undeveloped stage; **(B)** pre-vitellogenic stage; **(C)** early vitellogenic stage; **(D)** late vitellogenic stage; **(E)** mature stage. Fc, follicle cell; Oc, oocyte; Og, oogonium; N, nuclear; Nu, nucleolus. The arrows indicate different types of cells from various stages of ovaries.

**Figure 5:**
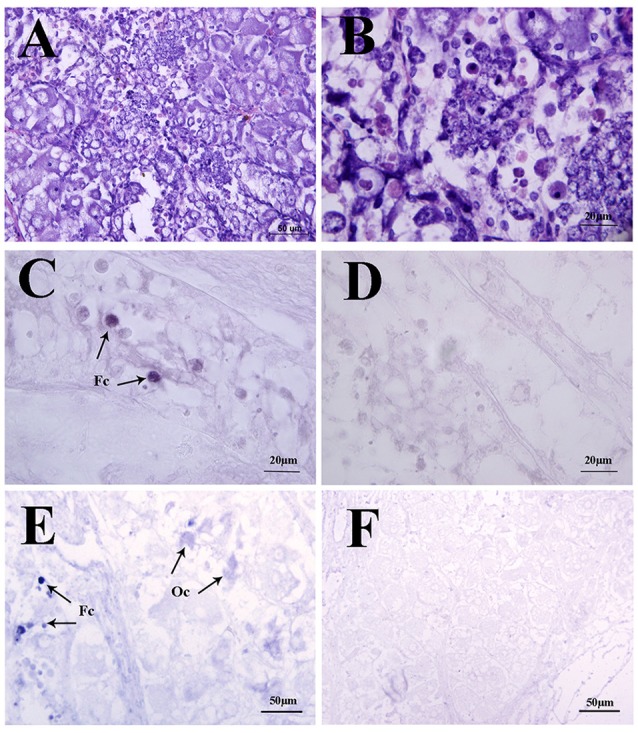
Localization of *Sp-sNPF* and *Sp-sNPFR* mRNA by *in situ* hybridization in the mud crab ovaries at the pre-vitellogenic stage. **(A, B)** Hematoxylin and eosin (HE) staining in the ovaries. Arrows indicate the specific *Sp-sNPF* **(C)** and *Sp-sNPFR* **(E)** mRNA signals with the antisense riboprobes in ovaries. Sense riboprobes of *Sp-sNPF* **(D)** and *Sp-sNPFR* **(F)** were used as the negative control. Fc, follicle cell; Oc, oocyte.

**Figure 6:**
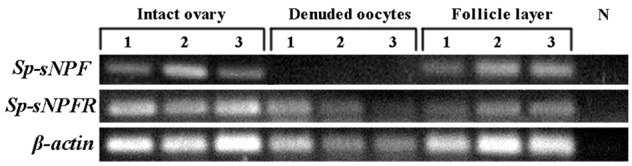
Intraovarian distribution of *Sp-sNPF* and *Sp-sNPFR* mRNA in a full-grown ovary. RT-PCR detection of *Sp-sNPF* and *Sp-sNPFR* gene expression in different compartments of full-grown ovaries (follicle layer and denuded oocyte). *Sp-sNPF* was exclusively detected in follicle layer, whereas *Sp-sNPFR* was expressed in both denuded oocytes and follicle layer. β*-actin* as the housekeeping gene.

In the mud crab, ovary consists of oocytes and follicle layers surrounding the oocytes (Figure 4). Histological study showed that oocytes were larger in size than the follicle cells at pre-vitellogenic stage (Figures 5A,B). *In situ* hybridization showed that the positive signals of *Sp-sNPF* mRNA were located on the follicle cells (Figure 5C) and *Sp-sNPFR* was detected in both oocytes and follicle layer cells (Figure 5E). No signal was detected when the sense riboprobes of *Sp-sNPF* (Figure 5D) and *Sp-sNPFR* (Figure 5F) were used. Results of RT-PCR matched the findings of *in situ* hybridization (Figure 6).

### Oocytes were directly activated by *Sp*-sNPFs

**Figure 7:**
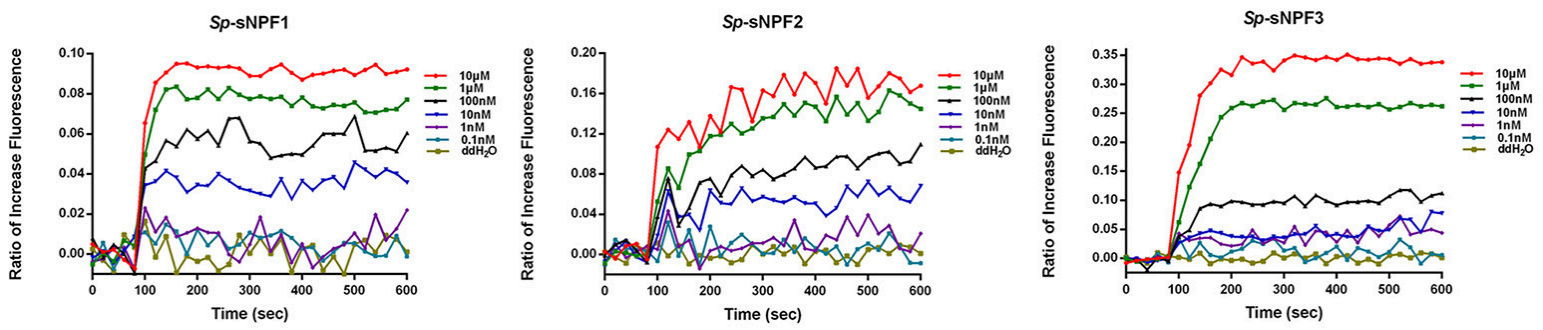
Intracellular Ca^2+^ mobilization of oocytes was induced by *Sp-*sNPF peptides. *Sp-*sNPF peptides evoked intracellular Ca^2+^ flux in a ligand concentration-dependent manner in oocytes. All events were performed in at least three independent experiments.

Based on our finding that *Sp-sNPFR* mRNA was detected in denuded oocytes (Figure 6), we employed Ca^2+^ mobilization technology to investigate whether oocytes were activated by *Sp*-sNPFs. As shown in Figure 7, oocytes exhibited a dose-dependent response in the intracellular Ca^2+^ mobilization when challenged with different concentrations of *Sp*-sNPF peptides.

### Effect of *Sp*-sNPFs on oocyte maturation

**Figure 8:**
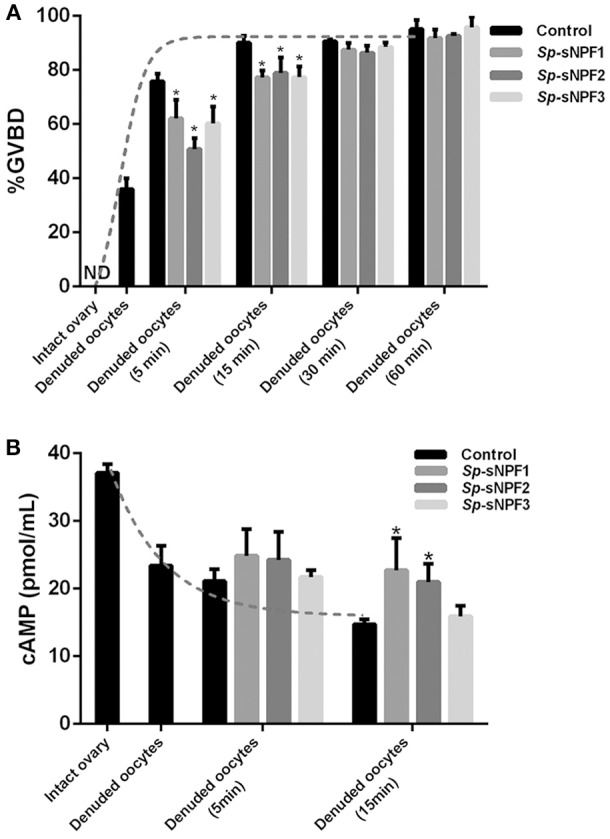
Inverse effects of *Sp-*sNPF peptides on maturation of denuded oocytes. **(A)** *Sp*-sNPF peptides suppressed GVBD rate in denuded oocytes. Denuded oocytes were incubated in the presence or absence of *Sp*-sNPF peptides (5 μM) for 0, 5, 15, 30, and 60 min before scoring GVBD oocytes. GVBD was inhibited by *Sp*-sNPF peptides at 5 and 15 min of incubation. The dotted line represents the GVBD rate trend of denuded oocytes which were isolated from an intact ovary in the control group. **(B)** cAMP accumulation was measured with ELISA. The dotted line represents the cAMP accumulation trend of denuded oocytes, which were isolated from intact ovary, in the control group. All values are presented as mean ± SD (*n* = 3) from three independent experiments (*P < 0.05).

To explore the potential role of *Sp*-sNPF in oocyte maturation, denuded oocytes (GV stage) which were separated from the ovaries were chosen. As indicated in Figure 8A, denuded oocytes underwent GVBD fast and over 90% oocytes exhibited GVBD after 15 min in the control group. However, the time of GVBD ratio was delayed to 30 min in oocytes treated with 5 μM *Sp*-sNPF peptides (Figure 8A). Moreover, the GVBD ratio of oocytes was decreased in oocytes incubated with *Sp*-sNPF1, *Sp*-sNPF2, and *Sp*-sNPF3 for 5 min by 18, 33, and 21%, respectively. GVBD ratio of oocytes was decreased by 14, 12, and 14%, respectively, when *Sp*-sNPF1, *Sp*-sNPF2, and *Sp*-sNPF3 were used for incubation at 15 min (Figure 8A). We also observed a higher level of cAMP in oocytes incubated with 5 μM *Sp*-sNPF peptides (Figure 8B). This was in contrast to control oocytes where the cAMP level was reduced during the GVBD process (Figure 8B).

### Effect of *Sp*-sNPFs on expression of *Sp-Vg* and *Sp-VgR in vivo*

**Figure 9:**
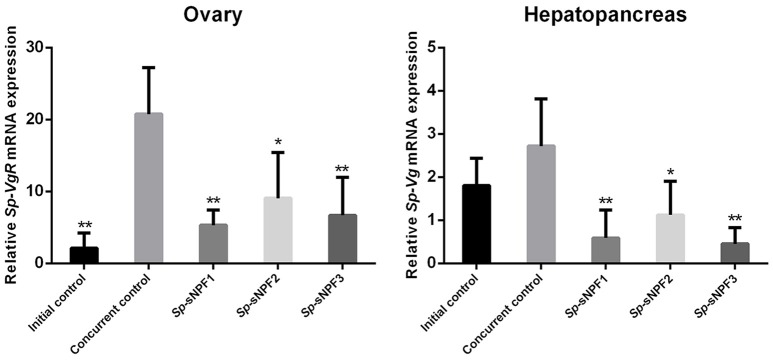
Inhibitory effects of *Sp-*sNPF peptides on the expression of *Sp-Vg* and *Sp-VgR in vivo*. Hepatopancreas and ovaries were sampled at 10 days post-injection of *Sp-*sNPF peptides for detecting the expression levels of *Sp-Vg* and *Sp-VgR*. The relative expression levels were normalized to β*-actin*. All data are presented as mean ± SD (*n* = 4) from a representative experiment. *P < 0.05, **P < 0.01 vs. concurrent control.

Our previous study demonstrated that the expression of *Sp-sNPF* decreased continuously during vitellogenesis (44). In this study, we investigated the potential role of sNPF in vitellogenesis by analyzing the effect of injected synthetic *Sp*-sNPF peptides on the expression of *Sp-Vg* and *Sp-VgR* in hepatopancreas and ovaries derived from pre-vitellogenic females. We found that injection of *Sp*-sNPF1, *Sp*-sNPF2, and *Sp*-sNPF3 significantly inhibited *Sp-Vg* expression in hepatopancreas by 78, 59, and 83%, respectively. The expression level of *Sp-VgR* in ovaries was decreased by 74%, 56%, and 68%, in the presence of *Sp*-sNPF1, *Sp*-sNPF2, and *Sp*-sNPF3 respectively (Figure 9).

## Discussion

sNPFs have been identified from a broad range of arthropods (27). Since the discovery of the first sNPFR, a GPCR with structural similarity to NPY2R, in *D. melanogaster* (58), some sNPFRs have been identified in insects (33, 41, 59–68). Although a large number of sNPFs have been characterized in different crustacean species (44–50), no sNPFR has been identified in crustaceans. In the present study, analyses of sequence similarity in transcriptome database revealed a candidate sNPFR annotated as a NPY-like receptor (NPY2R) in *S. paramamosain*. In addition, the ligand-receptor activation of signaling demonstrated that the candidate sNPFR was activated by three synthetic *S. paramamosain* sNPF peptides, *Sp*-sNPF1, *Sp*-sNPF2, and *Sp*-sNPF3, in a dose-dependent manner, with EC_50_ of 1.2, 3.8, and 8.5 μM respectively and which resulted in cAMP accumulation. All other tested peptides with the RFamide C-terminus did not elicit a cAMP response. Therefore, we designated the candidate sNPFR as the *S. paramamosain* sNPF receptor (*Sp*-sNPFR). To our knowledge, this receptor is the first crustacean sNPF receptor that has been characterized in this study.

To date, several insect sNPF receptors have been cloned and characterized (33, 41, 59–68). Among these receptors, the sNPFRs from *D. melanogaster* (67, 68), *A. gambiae* (60), *B. mori* (41, 61), *S. gregaria* (33), *A. aegypti* (62), *S. invicta* (63), *G. m. morsitans* (64), and the oriental fruit fly, *Bactrocera dorsalis* (Hendel) (65) have been deorphanized by ligand-receptor activation of signaling in a heterologous cell expression system, with EC_50_ values in the nanomolar range. Compared to the high sensitivity of insect sNPFRs, *Sp*-sNPFR in our study had a relatively higher half-maximal effective concentration (EC_50_) in the HEK293T expression system. Likewise, the intracellular Ca^2+^ concentration of *S. paramamosain* oocytes were mobilized by relatively high concentrations of synthetic *Sp*-sNPF peptides *in vitro*. Nonetheless, our findings are similar to a study which showed that a short neuropeptide F-related receptor from the mollusk, *Crassostrea gigas* exhibited EC_50_ value in the micromolar range, similar to our results (69).

Studies in insect sNPFRs revealed that *Si*-sNPFR is localized in the ovary (43) suggesting that sNPF may directly act on ovary. As *S. invicta* sNPF was detected in the nervous system, authors speculated that sNPF may act as a neurohormone on the ovaries (43). In our previous study (44), we found a higher level of *Sp-sNPF* transcript in the ovary of *S. paramamosain* suggesting that *Sp*-sNPF may function as an autocrine/paracrine factor within the ovary.

Our results showed that *Sp-sNPF* mRNA was exclusively localized in the follicle cells, whereas *Sp-sNPFR* was present in both follicle cells and oocytes. These results implied that *Sp*-sNPF may bind *Sp*-sNPFR via an autocrine/paracrine way. The localization of *Sp*-sNPF and *Sp*-sNPFR is consistent with the intraovarian expression pattern of *S. paramamosain* bone morphogenetic protein (*Sp*-BMP) and its receptors (54). BMP belongs to the transforming growth factor β (TGF-β) superfamily, classical ovarian local autocrines/paracrines factors (70, 71). In vertebrates, BMPs and their receptors have a different localization, and are speculated to have an autocrine/paracrine regulatory mechanism in the ovary (72–75). The distribution of *Sp*-sNPF and *Sp*-sNPFR suggested that there may be a similar autocrine/paracrine regulatory mechanism mediated by *Sp*-sNPF in the ovary for follicle cells to signal the follicle cells/oocytes. Finally, the finding that the *Sp*-sNPF peptides elicited a dramatic increase in intracellular Ca^2+^ is consistent with its proposed role as an intraovarian regulatory factor through an autocrine/paracrine regulatory mechanism.

When FG oocytes were separated from the follicle cells, they underwent spontaneous maturation with almost all oocytes exhibiting GVBD in 15 min. Meanwhile, the cAMP level in the oocytes decreased significantly. In contrast, when *Sp*-sNPF peptides were added to the culture medium, GVBD rate of oocytes was significantly reduced, while the cAMP levels were partly reversed. This phenomenon is in agreement with the previous reports in zebrafish where removal of the follicle layers significantly increased in spontaneous maturation of the oocytes (76, 77). These findings suggest that surrounding follicle layer cells may play a similar role in both vertebrates and invertebrates in inhibiting oocyte maturation. The secretory factors that may be responsible for this inhibitory effect of follicle cells on oocyte maturation have not been fully explored. Estradiol (E_2_) and Pituitary Adenylate Cyclase Activating Polypeptide (PACAP) were identified as two such factors where E_2_ partly inhibited the spontaneous maturation of the denuded oocytes via GPER, a membrane receptor located in the oocytes (76), and PACAP had a similar inhibitory effect on oocyte maturation (77). *Sp*-sNPF may be one such factor that suppresses oocyte maturation by binding to *Sp*-sNPFR to promote cAMP accumulation. However, like E_2_ and PACAP, the inhibition of *Sp*-sNPF was incomplete suggesting that other factors could be involved in this regulatory mechanism.

To provide further clues to elucidate the importance of *Sp*-sNPF in *S. paramamosain* ovary, we performed *in vivo* experiments. In contrast to the previous reports in *L. migratoria* (35, 36), injection of *Sp*-sNPF peptides significant suppressed the expression of *Sp-VgR* in the pre-vitellogenic stage ovary in our study. VgR, located in the oocytes, has been regarded as a key receptor in the ovary which mediates the release of vitellogenin from hepatopancreas in crabs (78). Considering the correlation of Vg/VgR system, we also examined the expression level of *Sp-Vg* in hepatopancreas when the crabs were injected with *Sp*-sNPF peptides. In agreement with the expression pattern of *Sp-VgR, Sp*-sNPF peptides exhibited a similar inhibitory effect on *Sp-Vg* mRNA expression. The results suggest that *Sp*-sNPF may be an inhibitor of vitellogenesis.

In summary, this study shows the *S. paramamosain* orphan receptor NPY2R is a receptor for the neuropeptide *Sp*-sNPF (*Sp*-sNPFR). To our knowledge, this is the first sNPFR reported in crustaceans. Furthermore, the tissue and cellular localization of *Sp*-sNPF/*Sp*-sNPFR and the signal transduction are consistent with its proposed function as an intraovarian regulatory factor inhibiting vitellogenesis and oocyte maturation through an autocrine/paracrine regulatory mechanism. This research provides a new insight into the sNPF functions in reproduction.

## Author contributions

CB: Conceptualization, data curation, software, methodology, writing-original draft, project administration; YY: Conceptualization, data curation, methodology, writing-review, and editing; HH: Supervision; HY: Conceptualization, supervision, funding acquisition.

### Conflict of interest statement

The authors declare that the research was conducted in the absence of any commercial or financial relationships that could be construed as a potential conflict of interest.
